# Desmoplastic small round cell tumor cancer stem cell-like cells resist chemotherapy but remain dependent on the EWSR1-WT1 oncoprotein

**DOI:** 10.3389/fcell.2022.1048709

**Published:** 2022-11-25

**Authors:** Justin W. Magrath, Hong-Jun Kang, Alifiani Hartono, Madelyn Espinosa-Cotton, Romel Somwar, Marc Ladanyi, Nai-Kong V. Cheung, Sean B. Lee

**Affiliations:** ^1^ Department of Pathology and Laboratory Medicine, Tulane University School of Medicine, New Orleans, LA, United States; ^2^ Department of Pediatrics, Memorial Sloan Kettering Cancer Center, New York, NY, United States; ^3^ Department of Pathology and Laboratory Medicine, Memorial Sloan Kettering Cancer Center, New York, NY, United States

**Keywords:** DSRCT, pediatric cancer, cancer stem cells, chemoresistance, sarcoma

## Abstract

Desmoplastic Small Round Cell Tumor (DSRCT) is a rare and aggressive pediatric cancer driven by the *EWSR1-WT1* fusion oncogene. Combinations of chemotherapy, radiation and surgery are not curative, and the 5-years survival rate is less than 25%. One potential explanation for refractoriness is the existence of a cancer stem cell (CSC) subpopulation able escape current treatment modalities. However, no study to-date has examined the role of CSCs in DSRCT or established *in vitro* culture conditions to model this subpopulation. In this study, we investigated the role of stemness markers in DSRCT survival and metastasis, finding that elevated levels of *SOX2* and *NANOG* are associated with worse survival in sarcoma patients and are elevated in metastatic DSRCT tumors. We further develop the first *in vitro* DSRCT CSC model which forms tumorspheres, expresses increased levels of stemness markers (*SOX2*, *NANOG*, *KLF4*, and *OCT4*), and resists doxorubicin chemotherapy treatment. This model is an important addition to the DSRCT tool kit and will enable investigation of this critical DSRCT subpopulation. Despite lower sensitivity to chemotherapy, the DSRCT CSC model remained sensitive to knockdown of the *EWSR1-WT1* fusion protein, suggesting that future therapies directed against this oncogenic driver have the potential to treat both DSRCT bulk tumor and CSCs.

## Introduction

Desmoplastic Small Round Cell Tumor (DSRCT) is a rare, aggressive form of pediatric cancer that most commonly affects adolescents and young adults (Gerald and Rosai, 1989; Lal et al., 2005; Honoré et al., 2019). DSRCT typically presents in the abdominal or pelvic region and is characterized by nests of malignant cells with a high nuclear to cytoplasmic ratio surrounded by fibrous stroma (Gerald et al., 1995). Intraperitoneal metastases are present in up to 90% of cases at diagnosis and extraperitoneal metastases are present in 25%–43% of cases, contributing to low survival rates (Lal et al., 2005; Honoré et al., 2019). The tumor cell of origin remains unknown and intriguingly DSRCT tumors stain positive for proteins normally found in a diverse range of tissues including epithelial, mesenchymal, muscular, and neural tissue (Chang, 2006).

DSRCT is caused by the t(11;22)(p13;q12) chromosomal translocation which fuses the N-terminal domain of Ewing sarcoma breakpoint region 1 gene, *EWSR1* (ch 22), to the C-terminal domain of Wilms tumor 1 gene, *WT1* (ch 11) (Ladanyi and Gerald, 1994; Gerald et al., 1995; Gerald and Haber, 2005). This novel fusion protein encodes an aberrant transcription factor whereby zinc fingers 2–4 of *WT1* direct the N-terminal low complexity domain (LCD) of *EWSR1* to alter gene expression and ultimately leads to carcinogenesis (Gerald and Haber, 2005). While many tumors contain genes with recurrent mutations that can be targeted therapeutically, recent studies have identified few recurrent mutations in DSRCT and no targeted therapies have yet been developed (Shukla et al., 2012; Devecchi et al., 2018). Current treatment consists of a combination of surgery, radiotherapy, and chemotherapy, commonly with the P6 regimen (Lal et al., 2005; Hayes-Jordan et al., 2016; Bulbul et al., 2017; Subbiah et al., 2018; Honoré et al., 2019). However, despite these interventions, the prognosis for DSRCT remains poor with a 5-years survival rate of only 15%–25% (Kushner et al., 1996; Lal et al., 2005; Subbiah et al., 2018; Honoré et al., 2019).

One potential explanation for the lack of therapeutic efficacy against DSRCT is the existence of a subpopulation of cells known as tumor initiating cells (TICs) or cancer stem cells (CSCs). CSCs have been identified in a variety of tumor types including leukemia, breast cancer, and glioblastoma (Bonnet and Dick, 1997; Al-Hajj et al., 2003; Bradshaw et al., 2016). Recently, CSCs were identified in the closely related Ewing sarcoma, which like DSRCT is characterized by chromosomal translocations involving *EWSR1* (most commonly the *EWSR1-FLI1* translocation) (Suva et al., 2009; Awad et al., 2010; Hotfilder et al., 2018). CSCs are defined by their ability to both self-renew and differentiate (Chang, 2016; Dagogo-Jack and Shaw, 2018). Further, they are chemoresistant and able to initiate tumors and form metastases (Kim et al., 2009; Chang, 2016; Zhao, 2016). *In vitro*, CSCs can be identified by their ability to form tumorspheres, the expression of stemness genes (such as *NANOG*, *SOX2*, and *OCT4*), slower growth, and chemoresistance (Walter et al., 2011; Kim and Alexander, 2014; Cole et al., 2020).

Given DSRCT’s positive staining for proteins from a variety of tissue types, resistance to treatment, and high rate of metastasis, we sought to investigate the potential role of CSCs in this tumor. We examined the association between stemness gene expression and survival in sarcomas and the expression of stemness genes in primary versus metastatic DSRCT tumors. Importantly, we developed a novel *in vitro* DSRCT tumorsphere model which exhibits many of the CSC properties. The DSRCT CSC-like population is slower-growing and chemoresistant yet maintains the ability to seed tumors *in vivo*. We further investigated the role of the *EWSR1-WT1* fusion protein in CSC-like cells. While a variety of cancers are dependent on fusion proteins, targeting the fusion protein is not always sufficient to eliminate the CSC population. While in Ewing sarcoma inhibiting the *EWSR1-FLI1* fusion protein with YK-4-279 reduced CSC clonogenicity, in chronic myeloid leukemia, inhibitors targeting the *BCR-ABL* fusion protein are unable to eliminate leukemia stem cells (LSCs) (Graham et al., 2002; Awad et al., 2010; Mustjoki et al., 2010; Thomas, 2012; Loscocco et al., 2019). This LSC persistence contributes to the need for kinase inhibitors to be taken throughout a patient’s life to prevent relapse. Intriguingly, the *EWSR1-WT1* fusion protein was expressed at a higher level in our DSRCT CSC model than normal adherent culture, and knockdown of the fusion protein reduced growth, decreased stemness, and led to apoptosis. These findings suggest that if a treatment targeting the *EWSR1-WT1* fusion protein is developed, it could eliminate both DSRCT bulk tumor and CSCs.

## Materials and methods

### Cell lines and culture conditions

JN-DSRCT-1, BER-DSRCT, and SK-DSRCT2 cell lines have been previously described and validated to harbor the defining *EWSR1-WT1* fusion (Nishio et al., 2002; Markides et al., 2013; Smith et al., 2022). Adherent culture: cells were grown on tissue culture (treated) plates in DMEM/F12 media supplemented with 10% FBS (Gibco), 2 mM L-Glutamine, 100 U/ml penicillin and 100 μg/ml streptomycin (ThermoFisher, Waltham, MA). Sphere culture: 4 * 10^6^ cells were seeded on non-treated plates (Costar^®^ 6-well Clear Not Treated Multiple, Corning) in a 1:1 mixture of DMEM/F12 and Neurobasal Media supplemented with 2 mM L-Glutamine, 100 U/ml penicillin, and 100 μg/ml streptomycin (ThermoFisher, Waltham, MA). Media was changed every 2 days.

### Patient-derived tumors

Three sets of patient-derived tumor pairs (primary/metastatic) were obtained from Memorial Sloan Kettering Cancer Center approved under IRB/Privacy Board 21-282. Each pair contained one tumor harvested from the abdominal region (primary) and one harvested from an extraperitoneal metastasis. Tumor sample information is included in Supplementary Table S1.

### Light microscopy

Light microscopy was performed with Nikon Eclipse 80i microscope using NIS-Elements software for image capture. Images of DSRCT cells in adherent culture were taken near confluence and in sphere conditions were taken at 4, 7, and 10 days after induction of sphere formation. Images for both conditions were taken at ×10 magnification.

### RNA isolation and real-time qPCR analysis

Total RNA from both tumor samples and cell culture was isolated with RNA-STAT60 (Tel-Test, Friendswood, TX). 500 ng of RNA was reverse transcribed to form cDNA using iScript cDNA Synthesis Kit (Bio-Rad, Hercules, CA). Relative transcript levels were analyzed by real-time qPCR using SYBR Green (SsoAdvanced Universal SYBR Green Supermix, Bio-Rad) and calculated by the comparative Ct method normalized against human ACTB (β-ACTIN) for cell culture and WT1 for patient tumor samples (as a control for tumor purity). Primers are listed in Supplementary Table S2.

### Protein isolation and Western blot analysis

Cell lysates were prepared in RIPA lysis buffer supplemented with complete EDTA-Free Protease Inhibitor Cocktail (Sigma-Aldrich), 1 mM NaF, and 2 mM Na_3_VO_4_. Proteins were resolved in 10% SDS-PAGE gels and transferred onto a 0.45 µm nitrocellulose membrane (Bio-Rad). Membranes were blocked with 5% non-fat milk and incubated with primary antibodies at 4°C overnight, followed by secondary antibodies LI-COR IRDye 800CW goat anti-Mouse (#926-32210, 1:15,000 dilution) or LI-COR IRDye 680RD goat anti-Rabbit (#926- 68071, 1:15,000 dilution) and scanned on LI-COR Odyssey CLx (Lincoln, NE). At least two independent immunoblots were performed for each experiment, with a representative immunoblot shown. Relative PARP cleavage was calculated using the formula:

$$Relative\;PARP\;Cleavage=\left(\frac{chemo\;treated\;cleaved\;PARP}{chemo\;treated\;uncleaved\;PARP}\right)-\left(\frac{ctrl\;cleaved\;PARP}{ctrl\;uncleaved\;PARP}\right)$$

Antibodies are listed in Supplementary Table S3.

### Cell growth and chemoresistance assays

Cell growth and chemoresistance assays were both performed using CCK-8 assay (Sigma-Aldrich) per the manufacturer’s directions. For cell growth assays, adherent and sphere cells were seeded in treated or non-treated 96-well plates with appropriate media at day 0 and CCK-8 was performed for the next 5 days (*n* = 6). For chemoresistance assays, adherent and sphere cells were seeded in treated or non-treated 96-well plates for 1 or 4 days respectively before drug addition. Cells were incubated with 10 nM to 10 μM of doxorubicin (Millipore Sigma), etoposide (Millipore Sigma), or cisplatin (Millipore Sigma) cells for 72 h before the CCK-8 assay was performed to assess viability. For all experiments, absorbance was measured using Clariostar microplate reader (BMG Labtech, Cary, NC).

### Cell cycle analysis

JN-DSRCT-1 and BER-DSRCT cells were grown in adherent culture (4 days) or sphere culture (4 or 7 days) (*n* = 3). Cells were fixed with 70% ethanol and stained with propidium iodide followed by flow cytometry. Cell-cycle analysis was performed using ModFit LT Software (Verity Software House).

### Xenografts in immune-deficient mice

All animal procedures were approved by the Tulane Institutional Animal Care and Use Committee. Male NOD-SCID-IL2Rγ-null (NSG) mice (6 weeks) were purchased (Jackson Laboratory, Bar Harbor, ME) and used for all xenograft studies. JN-DSRCT-1 and BER-DSRCT cells grown in adherent culture or sphere culture for 7 days were counted and mixed in a 1:1 ratio of media to Matrigel (Corning, Tewksbury, MA). 200 µl of cell mixture containing 1 * 10^6^ cells was subcutaneously injected into the lower flanks of NSG mice with adherent cells injected in the left flank and sphere cells injected in the right flank. Tumor volume was measured weekly with calipers and calculated as: length × (width)^2^ × 0.5, where length is the largest diameter and width is perpendicular to the length. Mice were sacrificed at 6 weeks post-injection. Tumors were harvested and weighed. Tumor fragments were stored in RNAlater for RNA isolation and fixed in formalin for immunohistochemistry analysis. Fixed tissues were embedded in paraffin, sectioned (5 μm), stained with H&E, and imaged (Nikon Eclipse 80i microscope; NIS-Elements software, Melville, NY).

### Generation of dox-inducible shRNA cell lines

Doxycycline (dox)-inducible LT3-GEPIR vector (Fellmann et al., 2013) was used to generate stable cell lines in BER-DSRCT and SK-DSRCT2 cells. Annealed oligonucleotides containing the shRNA sequence against WT1 3′UTR (*5′ GCA​GCT​AAC​AAT​GTC​TGG​TTA 3′*) was inserted into XhoI and EcoRI sites of the vector. Lentivirus was created by co-transfecting HEK293T cells with the LT3-GEPIR-shWT1 lentiviral vector and ViraPower lentiviral packaging mix (Invitrogen) using Lipofectamine3000 (Thermofisher Scientific). Viral supernatants were collected 48-, 72-, and 96-h post-transfection, and concentrated with LentiX-Concentrator (Takara Bio, San Jose, CA). BER-DSRCT and SK-DSRCT2 cells were transduced with LT3-GEPIR-shWT1 in the presence of polybrene (10 μg/ml) for 16 h. Cells were selected with puromycin (0.5 μg/ml for BER-DSRCT, 2 μg/ml for SK-DSRCT2) at 48 h post-transduction. Stable cell lines were validated by RT-qPCR and Western blot analyses with or without dox.

## Results

### SOX2 is highly expressed in desmoplastic small round cell tumor

**FIGURE 1:**
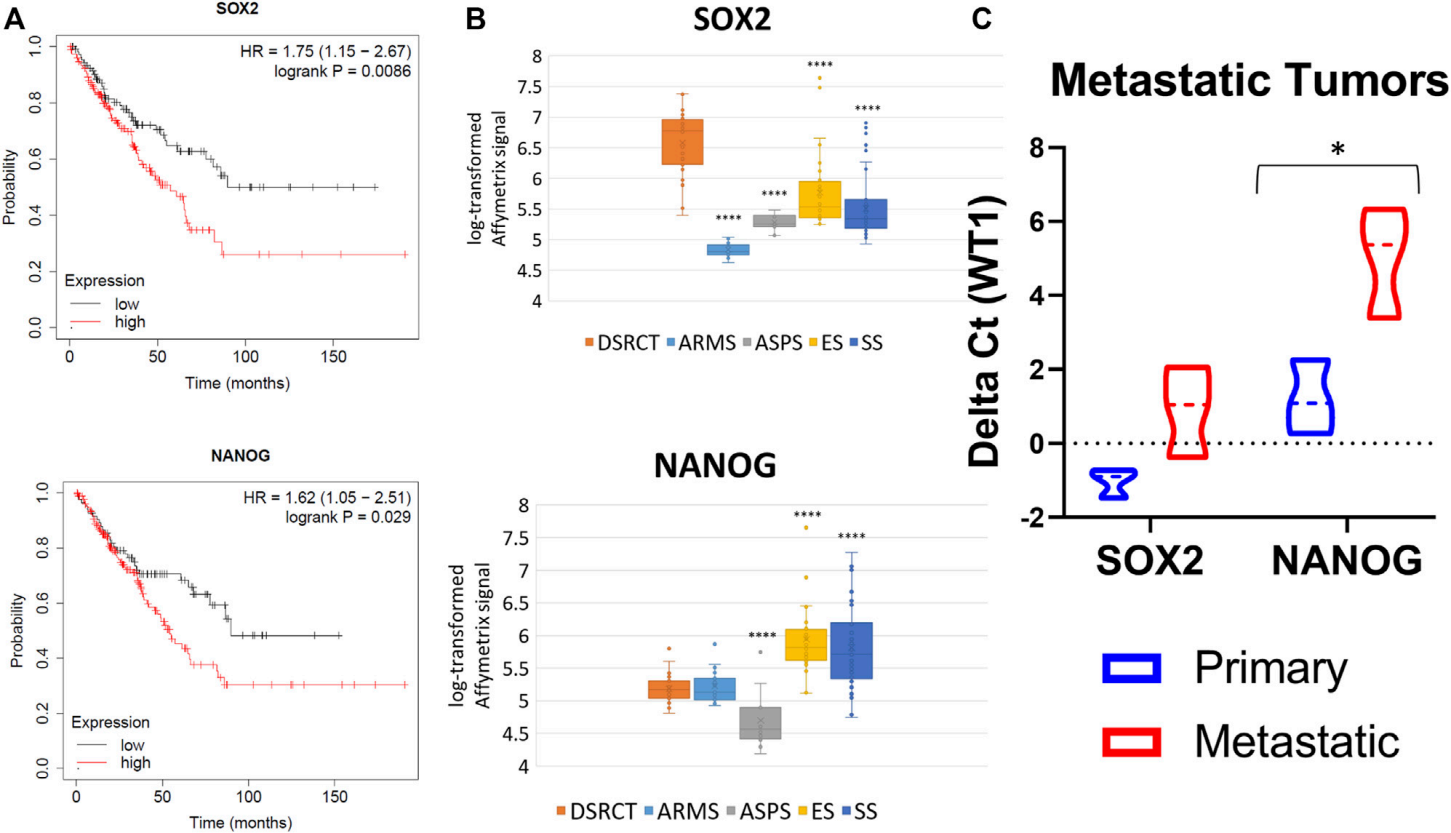
Stemness gene expression in DSRCT. **(A)** Kaplan-Meier curve of sarcoma patient survival based on *SOX2* (High *n* = 152, Low *n* = 107) and *NANOG* (High *n* = 103, Low *n* = 156) gene expression from KMplotter (total *n* = 259). **(B)** Relative transcript levels of *SOX2* and *NANOG* in DSRCT (*n* = 28) ARMS (*n* = 23), ASPS (*n* = 12), ES (*n* = 28), and SS (*n* = 46) primary tumors based on Affymetrix U133A expression array data. **(C)** Relative *SOX2* and *NANOG* mRNA expression in primary versus metastatic tumors assessed *via* RT-qPCR (*n* = 3, *p < 0.05, Student’s *t*-test).

To evaluate the clinical relevance of a DSRCT CSC population, we first examined the association between expression of stemness markers (*SOX2*, *NANOG*, *OCT4*, *KLF4*, *MYC*) and overall patient survival. Due to the rarity of DSRCT leading to a lack of available gene expression and correlated clinical data, we used Kaplan-Meier Plotter to examine patient survival in all sarcomas (Lánczky and Győrffy, 2021). Survival analysis revealed that higher expression of *SOX2*, *NANOG*, and *MYC* but not *OCT4* (*POU5F1*) or *KLF4* is associated with significantly reduced survival in sarcoma patients (Figure 1A; Supplementary Figure S1A). Using previously published gene expression data of fusion-positive sarcomas, we compared the expression of stemness genes in DSRCT (*n* = 28) to alveolar rhabdomyosarcoma (ARMS; *n* = 23), alveolar soft part sarcoma (ASPS; *n* = 12), Ewing sarcoma (ES; *n* = 28), and synovial sarcoma (SS; *n* = 46) (Filion et al., 2009). DSRCT had significantly higher expression of *SOX2* than the four other sarcoma types (Figure 1B). *NANOG* expression in DSRCT was higher than ASPS but lower than ES and SS (Figure 1B), while *OCT4* expression in DSRCT was higher than ARMS and ASPS but lower than ES (Supplementary Figure S1B). *KLF4* expression was highest in SS, while MYC expression was highest in ES (Supplementary Figure S1B). Because CSCs are thought to play an important role in metastasis, we reasoned that if a DSRCT CSC population exists, metastatic DSRCT tumors would likely express higher levels of stemness genes. RT-qPCR analysis was performed on three sets of primary versus metastatic DSRCT tumors to evaluate the expression of *SOX2* and *NANOG*, genes our previous analyses found are 1) associated with worse prognosis in sarcoma and 2) expressed at higher rates in DSRCT than at least one other type of fusion-positive sarcoma. The expression of *SOX2* and *NANOG* were both increased in metastatic versus primary DSRCT (*p* = 0.07 and *p* = 0.04 respectively) (Figure 1C). Together, these findings suggest a potential role for CSC-like cells in survival and metastasis of DSRCT and warranted further investigation.

### A novel culture condition induces cancer stem cell-like characteristics in desmoplastic small round cell tumor

**FIGURE 2:**
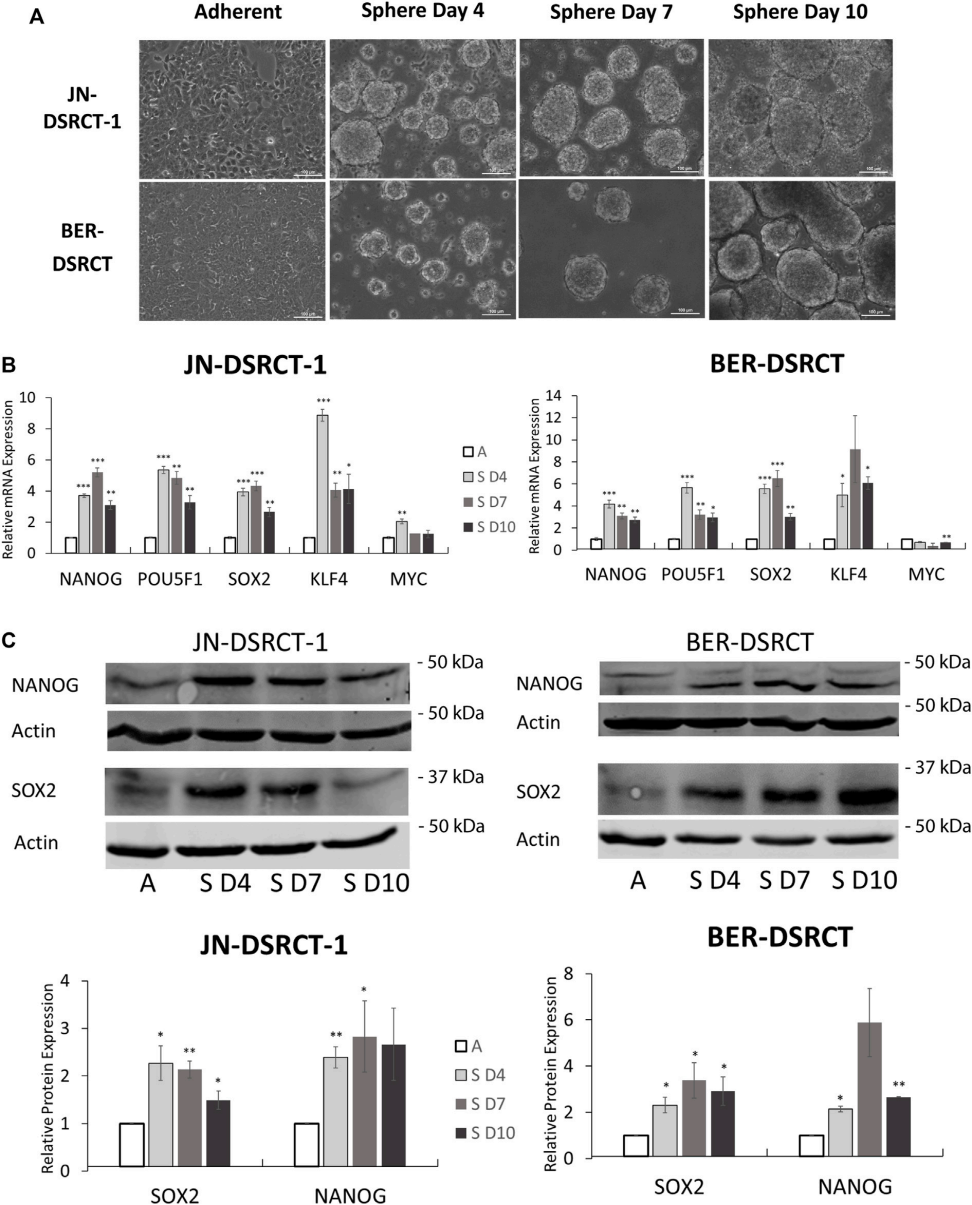
Tumorsphere formation increases stemness expression *in vitro*. **(A)** Light microscopy images of JN-DSRCT-1 and BER-DSRCT cells grown in adherent culture or sphere culture for 4, 7, and 10 days. **(B)** Relative expression of *NANOG*, *POU5F1*, *SOX2*, *KLF4*, and *MYC* mRNAs in adherent culture **(A)**, 4 days of sphere culture (S D4), 7 days of sphere culture (S D7), or 10 days of sphere culture (S D10) assessed *via* RT-qPCR (*n* = 3, *p < 0.05, **p < 0.01, ***p < 0.001, Student’s *t*-test). **(C)** Western blot of *NANOG* and *SOX2* protein expression in adherent culture or 4, 7, or 10 days of sphere culture. Relative protein expression from independent biological replicates normalized to adherent expression using ImageJ is shown in lower panels (*n* ≥ 3, Error bars = SE, *p < 0.05, **p < 0.01).

*In vitro* tumorsphere formation is a hallmark of CSCs and a method of CSC enrichment. However, to date no study has demonstrated tumorsphere formation in DSRCT. To establish a DSRCT CSC-like model, the two commonly available DSRCT cell lines, JN-DSRCT-1 and BER-DSRCT, were cultured in a variety of conditions with different media, FBS composition, growth factors, attachment surface, and supplements, resulting in a novel culture condition (1:1 mixture of DMEM/F12 and Neurobasal media in non-treated plates) that enabled consistent tumorsphere formation in both cell lines (Figure 2A). Interestingly, growth factors and supplements, including EGF, FGF, B12, and N2, were dispensable, while the presence of FBS prevented tumorsphere formation. Tumorspheres clearly formed by day 4 in the novel culture condition and continued to increase in size from days 4–10.

Having established culture conditions that enable the formation of tumorspheres, gene expression changes were examined. Tumorsphere formation led to significant and robust increases in *NANOG*, *OCT4*, *SOX2*, and *KLF4* mRNA expression at 4-, 7-, and 10-days post tumorsphere induction when compared to adherent culture controls (Figure 2B). Gene expression increases varied between 3- to 15-fold. The highest expression of *NANOG* and *OCT4* in BER-DSRCT were achieved at 4 days post-induction, while the highest expression of *SOX2* in BER-DSRCT as well as the highest expression of all four genes in JN-DSRCT-1 were achieved at 7 days post-induction. The one stemness gene examined that did not show consistent increases was *MYC*. *MYC* expression slightly increased at day 4 relative to adherent control in JN-DSRCT-1 and slightly decreased at day 10 relative to adherent control in BER-DSRCT, but remained relatively similar at all other timepoints. Western blot of sphere and adherent culture samples from the same timepoints demonstrated increased expression of *NANOG* and *SOX2* at the protein level in both JN-DSRCT-1 and BER-DSRCT (Figure 2C). In alignment with our RT-qPCR data, the most robust protein expression was observed at days 4 and 7 post-induction. While in JN-DSRCT-1 we observed a single NANOG band *via* Western blot, in BER-DSRCT two NANOG bands were observed. BER-DSRCT cells showed not only an increase in NANOG expression, but also a switch from a higher molecular weight NANOG band to a lower molecular weight band, potentially suggesting an alteration in post-translational modification.

To further validate our novel CSC-like culture condition, we applied the same media to a third, recently established DSRCT cell line (SK-DSRCT2) (Smith et al., 2022). Similar to JN-DSRCT-1 and BER-DSRCT, SK-DSRCT2 cells were able to form spheres that increased in diameter over time (Supplementary Figure S2A). SK-DSRCT2 cells grown in sphere versus adherent conditions also expressed increased levels of stemness markers at the RNA and protein levels (Supplementary Figures S2B,C). Notably, NANOG expression was absent at the transcript and protein levels in SK-DSRCT2 in both adherent and CSC-like culture conditions.

### Desmoplastic small round cell tumor cancer stem cell-like cells are chemoresistant

**FIGURE 3:**
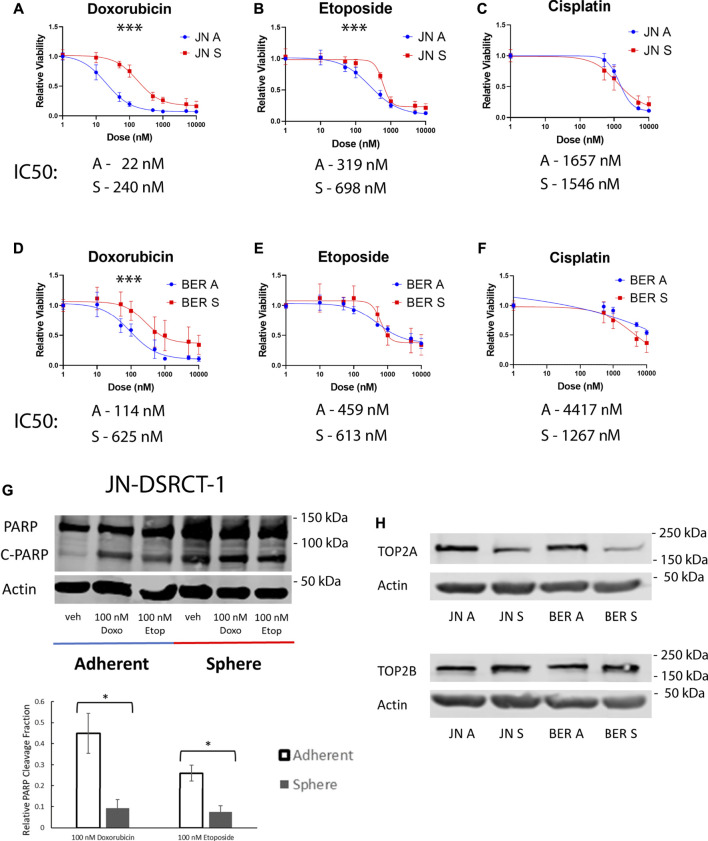
DSRCT CSC-like cells are resistant to chemotherapy. **(A–C)** Relative viability of JN-DSRCT-1 cells grown in adherent or sphere culture and treated with doxorubicin **(A)**, etoposide **(B)** or cisplatin **(C)** for 72-h. **(D–F)** Relative viability of BER-DSRCT cells grown in adherent or sphere culture and treated with doxorubicin **(D)**, etoposide **(E)** or cisplatin **(F)** for 72-h (***p < 0.001, ANOVA). **(G)** Western blot of PARP and cleaved PARP after 24 h treatment with vehicle, 100 nM doxorubicin, or 100 nM etoposide in adherent or sphere culture conditions in JN-DSRCT-1 (*n* = 3, *p < 0.05). **(H)** Western blots of *TOP2A* and *TOP2B* protein expression in JN-DSRCT-1 and BER-DSRCT cells grow in adherent (A) or sphere (S) culture.

Chemoresistance to the common DSRCT therapeutics etoposide, doxorubicin, and cisplatin was next examined. Etoposide and doxorubicin are part of the P6 chemotherapy regimen, which is the standard of care for DSRCT and includes etoposide, doxorubicin, vincristine, cyclophosphamide, and ifosfamide (Honoré et al., 2019). Cisplatin on the other hand has been used *via* hyperthermic intraperitoneal chemotherapy for the treatment of DSRCT (Hayes-Jordan et al., 2010; Hayes-Jordan et al., 2018). However, despite the use of these therapies, DSRCT survival remains low, potentially as a result of the existence of a CSC-like population. DSRCT cells in adherent or sphere culture conditions were treated for 72-h with doses of each drug ranging from 10 nM to 10 µM. The CCK-8 assay was performed to examine relative viability. Cells in sphere culture were less sensitive to treatment with doxorubicin and etoposide but not cisplatin as compared to cells in adherent culture (Figures 3A–F). Both cell lines showed statistically significant differences in viability between sphere and adherent culture for doxorubicin treatment, while for etoposide JN-DSRCT-1 showed a statistically significant difference but the difference for BER-DSRCT did not rise to statistical significance. These results not only demonstrate the chemoresistance of sphere DSRCT cells, but to our knowledge are the first *in vitro* examination of the efficacy of these three chemotherapeutics on DSRCT, despite their current clinical application. These data show that DSRCT cells are most sensitive to doxorubicin, followed by etoposide, and not very sensitive to cisplatin.

To further confirm the observed chemoresistance of DSRCT cells in CSC-like culture, cells in adherent and sphere culture were treated with 100 nM doxorubicin, 100 nM etoposide, or vehicle control for 24-h and protein was harvested. Western blot was used to examine PARP cleavage as a marker for apoptosis. We found that sphere culture cells have a higher baseline level of PARP cleavage as compared to adherent culture cells, potentially reflective of the harsher culture conditions (e.g., lack of FBS and anoikis). However, consistent with our previous results we found that sphere culture cells were less sensitive to treatment with doxorubicin or etoposide as demonstrated by a lower change in the relative PARP cleavage fraction compared to untreated controls. The differences in PARP cleavage fraction between sphere and adherent culture were statistically significant for both doxorubicin and etoposide treatment in JN-DSRCT-1 (Figure 3G) but not BER-DSRCT (Supplementary Figure S3). BER-DSRCT treatment with doxorubicin showed a similar trend as JN-DSRCT-1, while its PARP cleavage fraction with etoposide treatment showed higher cleavage for the sphere versus adherent state. While overall showing the chemoresistance of the sphere culture state, these results indicate potential variability between DSRCT cell lines and a more pronounced resistance of CSC-like cells against doxorubicin than etoposide.

Since doxorubicin and etoposide are both topoisomerase II poisons, one potential mechanism for chemoresistance is altered expression of topoisomerase II. Protein expression of the two forms of topoisomerase II (*TOP2A* and *TOP2B*) was examined *via* Western blot. In both JN-DSRCT-1 and BER-DSRCT cell lines, *TOP2A* expression was substantially lower in sphere culture than adherent culture (Figure 3H). Conversely, *TOP2B* expression was slightly higher in sphere culture than adherent culture (Figure 3H). Topoisomerase II poisons act by stabilizing DNA:TOP2 covalent complexes resulting in increased DNA damage and leading to cell death (Nitiss, 2009). In lymphoma, knockdown of *TOP2A* was found to reduce DNA damage and enable resistance to doxorubicin treatment (Burgess et al., 2008). By a similar mechanism reduced expression of *TOP2A* in the DSRCT CSC model may help explain resistance to doxorubicin and etoposide treatment.

### Desmoplastic small round cell tumor cancer stem cell-like cells are quiescent

**FIGURE 4:**
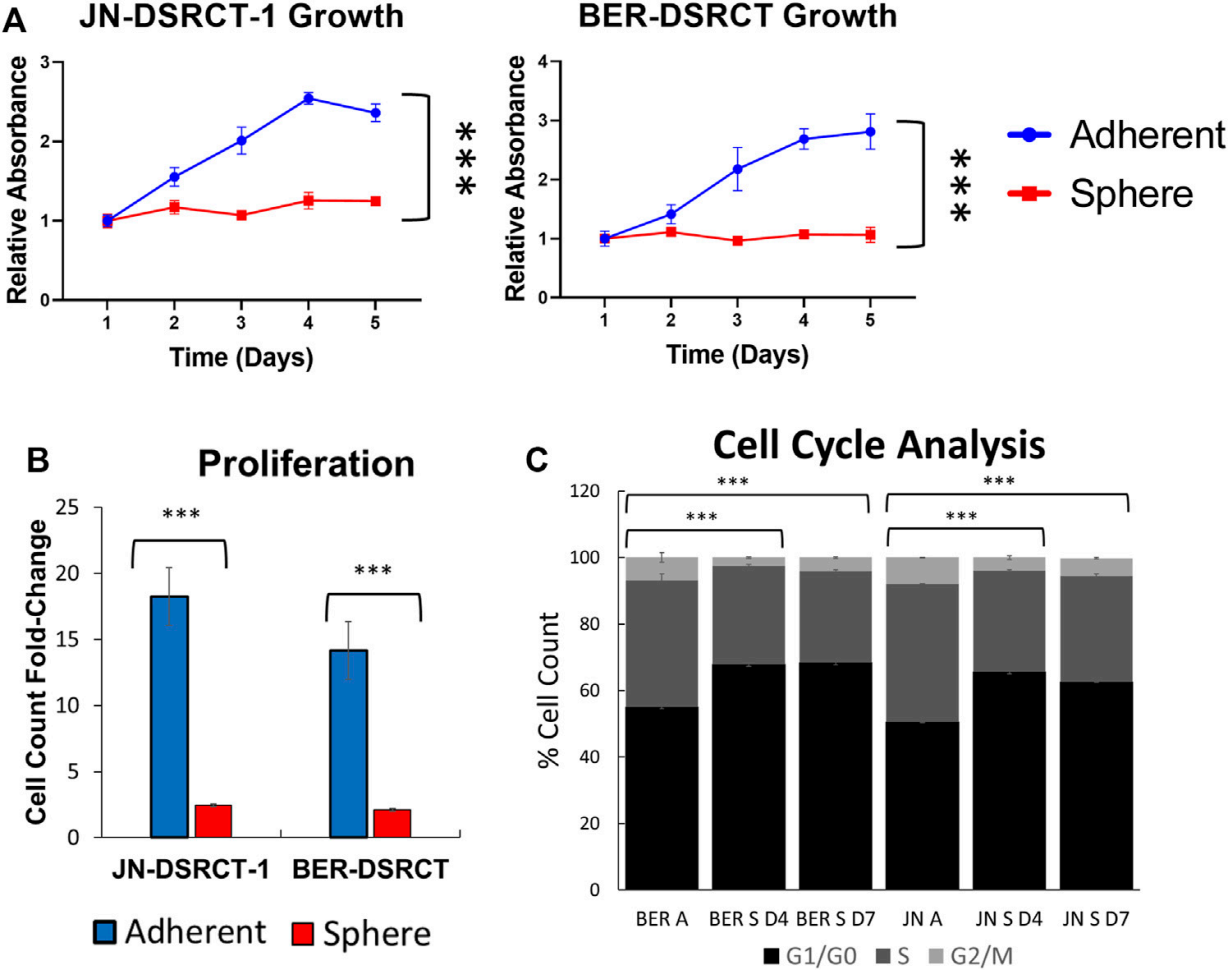
DSRCT CSC-like cells are quiescent. **(A)** Growth of JN-DSRCT-1 and BER-DSRCT cells in adherent or sphere culture conditions for 5 days as determined by CCK-8 (*** *p*-value < 0.001, ANOVA). **(B)** Proliferation of JN-DSRCT-1 and BER-DSRCT cells in adherent or sphere culture conditions for 5 days assessed as fold-change in cell count. **(C)** Cell cycle analysis of JN-DSRCT-1 and BER-DSRCT cells grown in adherent culture, 4 or 7 days of sphere culture.

A quiescent cell state marked by slow growth can reduce sensitivity to chemotherapeutics and contribute to CSC chemoresistance (Cole et al., 2020; Francescangeli et al., 2020). *TOP2A* expression is linked to cell proliferation and a more quiescent state could contribute to the observed lower expression levels in DSRCT CSC-like cells (Pommier et al., 2010). To investigate the growth rate of cells in the DSRCT tumorsphere model, JN-DSRCT-1 and BER-DSRCT cells were seeded in adherent or sphere culture and growth was monitored using the CCK-8 assay every 24 h for 5-days. While the adherent cells grew quickly throughout the 5-days period for both cell lines, cells under sphere culture condition showed stable metabolic activity throughout the 5-days period indicating a more quiescent state (Figure 4A). To validate this finding, we measured proliferation by seeding cells in sphere or adherent conditions and counting them after 5-days of growth. Significant differences in the cell count fold-change were found for both JN-DSRCT-1 and BER-DSRCT in adherent versus sphere conditions (18.2 ± 4.4 STD fold-change for adherent JN-DSRCT-1 versus 2.5 ± 0.2 STD fold-change for sphere JN-DSRCT-1 (*p* < 0.001) and 14.2 ± 1.9 STD fold-change for adherent BER-DSRCT versus 2.1 ± 0.2 STD fold-change for sphere BER-DSRCT (*p* < 0.001), Figure 4B). While the sphere culture cells showed significantly reduced proliferation, they did not completely stop dividing, with both cell lines more than doubling their cell count over the 5-day period. Cell cycle analysis further supported the existence of a quiescent state. Cells grown in sphere culture for 4 and 7 days were less likely to be in S and G2/M phase and more likely to be in G1/G0 phase as compared to adherent culture cells (Figure 4C).

### Desmoplastic small round cell tumor cancer stem cell-like cells form tumors *in vivo*

**FIGURE 5:**
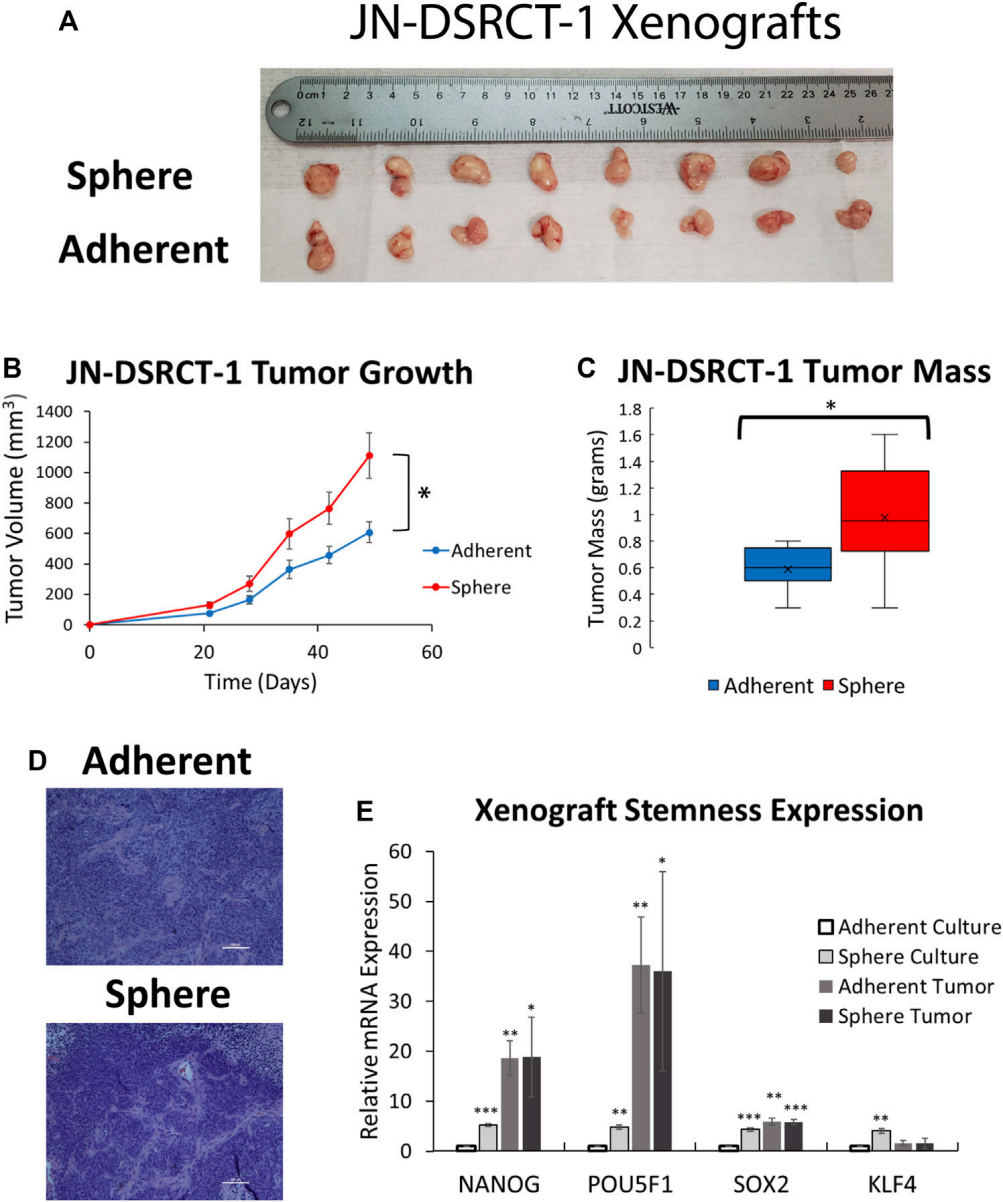
DSRCT CSC-like cells form tumors *in vivo*. **(A)** JN-DSRCT-1 sphere and adherent culture cells form tumors in 8/8 xenograft injections. **(B)** Tumor volume of JN-DSRCT-1 xenografts seeded from adherent or sphere culture cells (*p < 0.05, ANOVA). **(C)** Final mass of JN-DSRCT-1 xenografts seeded from adherent or sphere culture cells (*p < 0.05, Student’s *t*-test). **(D)** Representative H&E staining of JN-DSRCT-1 xenografts seeded from adherent or sphere culture cells. **(E)** Relative mRNA expression of *NANOG, POU5F1, SOX2, and KLF4* in JN-DSRCT-1 cells in adherent culture, sphere culture, xenograft tumors derived from adherent culture, or xenograft tumors derived from sphere culture assessed *via* RT-qPCR (*n* = 3, *p < 0.05, **p < 0.01, ***p < 0.001, Student’s *t*-test).

Given the reduced proliferation of sphere culture cells as compared to those in adherent culture, we next sought to examine whether these cells retained the ability to actively proliferate *in vitro* and form tumors *in vivo*. To test *in vitro* proliferation, DSRCT cells cultured in sphere culture for 7-days were plated back in adherent culture and growth was monitored using the CCK-8 assay. DSRCT cells from sphere culture were able to actively proliferate when plated in adherent culture conditions, with a similar rate of growth as cells maintained exclusively in adherent culture (data not shown). To examine *in vivo* tumor formation, JN-DSRCT-1 and BER-DSRCT cells were cultured in either adherent or sphere culture conditions for 7-days and injected subcutaneously into NOD.SCID.IL2Rγ-null (NSG) mice at 1 × 10^6^ cells per injection. Cells derived from sphere and adherent culture for both cell lines formed tumors in 8/8 injections by 5-weeks post injection (Figure 5A; Supplementary Figure S4A). JN-DSRCT-1 sphere-derived tumors showed greater size and mass than adherent-derived tumors (Figures 5B,C) while BER-DSRCT adherent-derived tumors had a larger tumor mass than and sphere-derived tumors (Supplementary Figures S4B,C). All tumors demonstrated the classic DSRCT histologic presentation of small round blue cells with desmoplasia (Figure 5D; Supplementary Figure S4D). The RNA expression of stemness markers *NANOG*, *OCT4*, *SOX2*, and *KLF4* was examined using RT-qPCR and compared to their expression as previously shown in adherent and sphere culture conditions *in vitro*. The expression of *NANOG*, *OCT4*, and *SOX2* in JN-DSRCT-1 tumors and all stemness markers in BER-DSRCT tumors was significantly greater than their expression in *in vitro* adherent culture conditions (Figure 5E; Supplementary Figure S4E). The expression of *NANOG* and *OCT4* in tumors was also many times greater than their expression in *in vitro* sphere culture conditions. Interestingly, with the exception of *NANOG* and *OCT4* in BER-DSRCT tumors, the expression of stemness genes was similar in sphere-derived and adherent-derived tumors. Given that sphere cells are more quiescent than adherent cells *in vitro*, these results showed that sphere cells are able to generate similar xenograft tumors as the actively proliferating adherent cells. Our results further demonstrated that adherent DSRCT cells, once grafted, were able to increase the expression of stemness genes and form tumors efficiently. Taken together with our previous results examining chemoresistance, this demonstrates that DSRCT CSC-like cells possess the two critical CSC characteristics of 1) chemoresistance and 2) tumor formation, suggesting the importance of eliminating this population to prevent tumor recurrence.

### EWSR1-WT1 is essential for the desmoplastic small round cell tumor cancer stem cell-like phenotype

**FIGURE 6:**
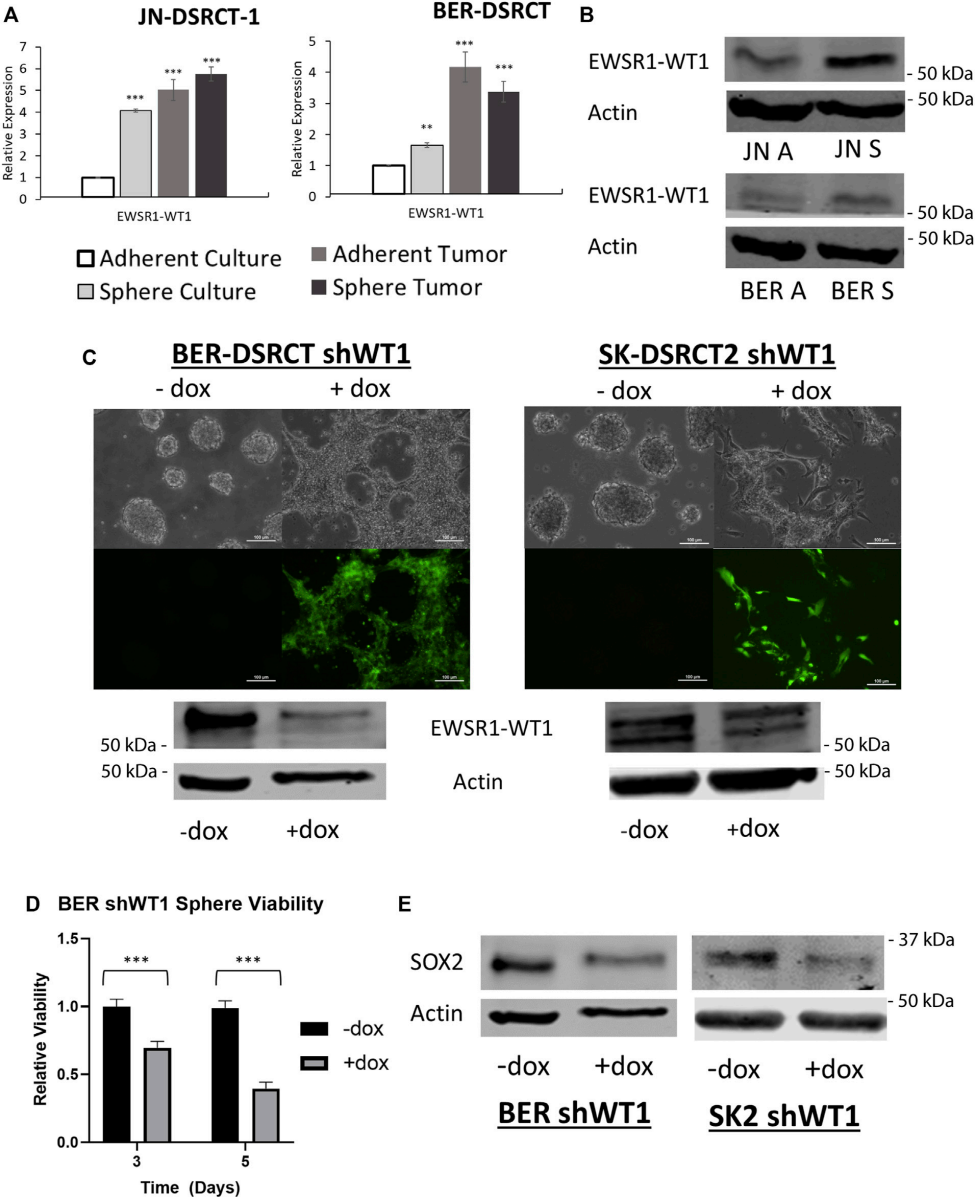
EWSR1-WT1 is necessary for DSRCT CSC characteristics. **(A)** Relative expression of *EWSR1-WT1* mRNA in JN-DSRCT-1 and BER-DSRCT cells in adherent culture, sphere culture, xenograft tumors derived from adherent culture, or xenograft tumors derived from sphere culture assessed *via* RT-qPCR (*n* = 3, *p < 0.05, **p < 0.01, ***p < 0.001, Student’s *t*-test). **(B)** Western blot of *EWSR1-WT1* protein levels in JN-DSRCT-1 and BER-DSRCT cells in adherent and sphere culture. **(C)** Dox-inducible shWT1 system induces selective expression of *EGFP* (as shown *via* microscopy) and knockdown of *EWSR1-WT1* protein (as shown *via* Western blot) in BER-DSRCT and SK-DSRCT2 cells only in the presence of dox. This knockdown reduced tumorsphere formation as shown *via* microscopy. **(D)** Relative viability of BER-DSRCT shWT1 in sphere culture for 3 and 5 days with or without dox addition (*n* = 4, ***p < 0.001, Student’s *t*-test). **(E)** Western blot of SOX2 protein levels in BER-DSRCT shWT1 and SK-DSRCT2 shWT1 cells in sphere culture for 7 days with or without dox addition.

Having established the DSRCT CSC-like model, we next utilized this model to examine the importance of the *EWSR1-WT1* fusion gene in DSRCT CSC-like cells. RNA expression of *EWSR1-WT1* was examined in JN-DSRCT-1, BER-DSRCT, and SK-DSRCT2 in both sphere and adherent culture as well as in xenograft tumors derived from these cultures. The expression of *EWSR1-WT1* was enriched 4-fold for JN-DSRCT-1, 1.7-fold for BER-DSRCT, and 1.3-fold for SK-DSRCT2 cells grown in sphere as opposed to adherent culture (Figure 6A; Supplementary Figure S5A). RNA expression of *EWSR1-WT1* was also upregulated in both sphere-derived and adherent-derived tumors as compared to *in vitro* adherent culture. Similarly, fusion protein expression *via* Western blot showed increased expression in sphere versus adherent cells (Figure 6B).

To determine whether *EWSR1-WT1* is necessary for tumorsphere formation, we employed our previously established doxycycline (dox)-inducible *EWSR1-WT1* knockdown system that utilizes shRNA targeting the 3′ UTR of *WT1* to reduce fusion gene expression (Hartono et al., 2022). Because native *WT1* is not expressed in DSRCT, shRNA targeting the 3′ UTR of *WT1* specifically silences the *EWSR1-WT1* fusion gene without a concern for *WT1* off-target effects (Hingorani et al., 2020). In addition to expressing shRNA targeting *WT1*, the dox-inducible system expresses EGFP controlled by the same promoter, leading to selective expression of EGFP when dox is added which enables easy verification of proper system functionality. BER-DSRCT and SK-DSRCT2 cells containing the dox-inducible sh*WT1* expression system cultured without dox did not express EGFP and were able to form tumorspheres (Figure 6C). When dox was added at the beginning of sphere culture induction, EGFP was expressed and cells failed to form tumorspheres (Figure 6C). When dox was added after four days of culture, when tumorspheres had already formed, dox addition led to EGFP expression and dissolution of tumorspheres (Supplementary Figure S5C). These observations suggest the *EWSR1-WT1* fusion protein is critical for both formation and maintenance of tumorspheres. RT-qPCR and western blot confirmed successful knockdown of *EWSR1-WT1* induced by dox added at sphere formation induction (Figure 6C; Supplementary Figure S5B). Knockdown of *EWSR1-WT1* reduced cell viability of BER-DSRCT CSC-like cells at 3- and 5-days post dox treatment (Figure 6D) while SK-DSRCT2 cells showed viability reductions 5 days after treatment (Supplementary Figure S5D). Fusion protein knockdown also reduced the expression of the stemness marker *SOX2* in both cell lines (Figure 6E) and *NANOG* in BER-DSRCT (Supplementary Figure S5E). An increase in *PARP* cleavage was observed in both cell lines after *EWSR1-WT1* knockdown in sphere culture for 7-days, validating our findings of reduced viability (Supplementary Figure S5F). Together, our findings suggest *EWSR1-WT1* is critical to tumorsphere formation and maintenance, stemness marker expression, and viability in the DSRCT CSC model.

## Discussion

Despite initial response, DSRCT typically becomes refractory to multimodal therapy, leading to an overall survival rate of only 15%–25% (Subbiah et al., 2018; Honoré et al., 2019). This poor survival rate could be explained by the existence of a CSC subpopulation that is able to resist chemotherapy, leading to tumor recurrence. However, no previous study has identified CSCs in DSRCT or established an *in vitro* DSRCT CSC model. In this study, we for the first time established a DSRCT CSC model which formed tumorspheres *in vitro*, had increased expression of stemness markers (*SOX2*, *NANOG, OCT4, KLF4*)*,* and was able to resist chemotherapy. Our findings that *SOX2* is highly expressed in DSRCT, associated with worse survival in sarcoma patients, and enriched in metastatic DSRCT tumors suggest the clinical relevance of DSRCT subpopulations with high *SOX2* expression and the importance of a model that enables their investigation.

Consistent with other *in vitro* CSC models, our novel DSRCT CSC-like model utilized low attachment cell culture plates and a defined media lacking serum (Ishiguro et al., 2017). Many CSC culture conditions also include supplements such as MSC stimulatory supplements for Ewing sarcoma CSCs and EGF, bFGF, and B27 for glioblastoma and rhabdomyosarcoma CSCs (Awad et al., 2010; Walter et al., 2011; Magrath et al., 2020). Intriguingly, these supplements, while not inhibitory, were dispensable for tumorsphere formation in DSRCT. This finding is concordant with a recent study that established the novel OV-054 DSRCT cell line and demonstrated the ability of this cell line to grow in suspension culture without the need for growth factor supplementation (Bleijs et al., 2021). Despite these differences in culture conditions, our DSRCT CSC-like model demonstrated similar increases in stemness markers as the CSCs in other cancer types, with stemness marker inductions ranging from 3- to 10-fold in comparison to adherent culture conditions (Walter et al., 2011; Magrath et al., 2020). Intriguingly, in the BER-DSRCT cell line, Western blot analysis demonstrated not only an increase in *NANOG* expression at the protein level, but also a switch in predominance from a higher molecular weight band to a lower molecular weight band. Eleven phosphorylation sites have been identified on *NANOG* and several have been shown to affect *NANOG*’s ability to reprogram cells to a more stem-like state (Brumbaugh et al., 2014; Saunders et al., 2017). Work by both Kim et al. (2014), Saunders et al. (2017) have found that phosphorylation can decrease the ability of *NANOG* to reprogram cells toward a stem-like state. Saunders et al. (2017) suggested this reprograming was stability independent, while Kim et al. (2014) found an effect of phosphorylation on *NANOG* protein stability. Our finding of an increase in the lower *NANOG* band in sphere culture conditions aligns with these previous studies and suggests dephosphorylation may explain the reduced molecular weight observed for *NANOG* in BER-DSRCT sphere culture cells.

It has been postulated that tumorsphere formation not only enriches for CSCs but may also be a better model of *in vivo* tumor conditions due to increased cell-to-cell interaction and the presence of hypoxia and nutrient gradients that are typical features of the tumor microenvironment (Ishiguro et al., 2017)*.* Gene expression analysis of breast cancer cells in xenograft, adherent culture, and spheroid culture by Hongisto et al. (2013) found that spheroid culture more closely mimics the gene expression profile of xenografts than adherent culture, suggesting it may be a better model for drug evaluation. While there were 2,428 differentially expressed genes between the xenograft tumor and adherent culture, the number of differentially expressed genes between xenograft tumor and spheroid culture was less than half as many (952 genes) (Hongisto et al., 2013). Similarly, we found that the expression of stemness markers *NANOG*, *SOX2*, *OCT4*, and *KLF4* as well as the *EWSR1-WT1* fusion gene in DSRCT xenograft models was more similar to their expression levels in our spheroid, CSC-like cells than adherent cells. The relevance of spheroid culture to drug evaluation was examined in a study by Kim and Alexander (2014) who found that spheroid cells were superior to adherent cells in predicting the response of xenograft tumors to chemotherapy. While adherent cells showed sensitivity to both doxorubicin and paclitaxel, the spheroid model accurately predicted the xenograft’s ability to respond to doxorubicin treatment but not treatment with paclitaxel (Kim and Alexander, 2014). Our novel DSRCT CSC-like model is therefore an important addition to the DSRCT tool kit and should be utilized to test the ability of potential therapeutics to target the DSRCT CSC population.

In the current study, we found that the DSRCT CSC model was less sensitive to doxorubicin and etoposide than adherent cells which may help to explain the clinical observation of initial response to chemotherapy frequently followed by DSRCT recurrence (Lal et al., 2005; Subbiah et al., 2018). Our findings further suggest downregulation of *TOP2A* as one potential mechanism of DSRCT resistance to chemotherapy. We did not find a difference in the sensitivity of DSRCT spheres and adherent cells to cisplatin treatment. Cells in both conditions showed limited sensitivity to cisplatin with a >5 μM cisplatin dose required to reduce viability relative to vehicle treated controls. This lack of DSRCT sensitivity to cisplatin is surprising since a number of clinical studies, including a recently published phase 2 clinical trial, have suggested that hyperthermic intraperitoneal cisplatin treatment improves survival in DSRCT (Hayes-Jordan et al., 2007; Hayes-Jordan et al., 2010; Hayes-Jordan et al., 2018). Despite the use of cisplatin clinically, this study is to our knowledge the first examination of DSRCT sensitivity to cisplatin *in vitro*. The discordance between our *in vitro* findings and reported clinical response could suggest both adherent and sphere *in vitro* culture conditions fail to mimic some aspect of DSRCT biology that is important to cisplatin sensitivity. Alternatively, this apparent discordance could suggest that the improvements in clinical survival attributed to cisplatin may have an alternative explanation. The phase 2 trial was non-randomized without negative controls and required prior response to chemotherapy as an inclusion criterion (Hayes-Jordan et al., 2018). Further, all surgeries were performed at one institution by the same two surgeons (Hayes-Jordan et al., 2018). While the resulting 3-year overall survival of 79% was superior to previous studies that lacked intraperitoneal cisplatin [55% by Lal et al. (2005)], this difference may be caused by other study variables including the skill of the surgeons, variation in presurgical chemotherapy regimens, and preselection of patients who respond to chemotherapy (Lal et al., 2005; Hayes-Jordan et al., 2018). Our finding that DSRCT has limited sensitivity to cisplatin *in vitro* is in-line with a retrospective study by Honoré et al. (2015) which did not find a significant improvement in survival with the addition of intraperitoneal cisplatin. Taken together, these discrepancies highlight the need for randomized controlled trials to definitively determine whether intraperitoneal cisplatin has clinical benefits in DSRCT.

The increased resistance of our DSRCT CSC model to doxorubicin and etoposide coupled with the lack of sensitivity to cisplatin suggest that alternative therapies able to specifically target DSRCT CSCs are urgently needed to improve DSRCT clinical outcomes. A high-throughput screen of 16,000 compounds in breast cancer stem cells identified the ionophore salinomycin as a potential CSC targeting therapy (Gupta et al., 2009). Salinomycin has further demonstrated CSC-targeting ability in colorectal cancer, glioblastoma, and chronic lymphocytic leukemia (Dong et al., 2011; Lu et al., 2011; Magrath and Kim, 2017). Other CSC-targeting therapies under investigation include monoclonal antibodies against CSC surface markers, for example adecatumumab targeting EpCAM in prostate cancer, and therapies that disrupt pathways enriched in CSCs, such as the Wnt, Notch, and Hedgehog pathways (Oberneder et al., 2006; Le et al., 2015; Norsworthy et al., 2019; Yang et al., 2020). Future work could utilize our novel DSRCT CSC model to examine gene expression alterations in the CSC-like subpopulation and identify targetable pathways for DSRCT CSCs. Another strategy for targeting the DSRCT CSC population may be by directly targeting the *EWSR1-WT1* fusion protein. While this strategy has been unable to eradicate leukemia stem cells in chronic myelogenous leukemia, a study by Awad et al. (2010) found that Ewing sarcoma CSCs were sensitive to inhibition by the small molecule YK-4-279 that targets the *EWSR1-FLI1* fusion protein (Graham et al., 2002). Utilizing a dox-inducible shRNA system to knockdown the *EWSR1-WT1* fusion gene in two DSRCT cell lines, we demonstrated that DSRCT CSC-like cells remain sensitive to fusion protein knockdown and that fusion protein knockdown reduces both sphere formation and expression of the stemness marker *SOX2*. A recent study by Gedminas et al. (2022) proposed lurbinectedin as an inhibitor of the *EWSR1-WT1* fusion protein in DSRCT. Our findings suggest that strategies targeting the *EWSR1-WT1* fusion including inhibitors and anti-sense oligonucleotides have the potential to eliminate not only bulk tumor but also the DSRCT CSC population.

## Data Availability

Publicly available datasets were analyzed in this study. This data can be found here: http://cbio.mskcc.org/public/sarcoma_array_data/.
