# Supplementary material for: Desmoplastic small round cell tumor cancer stem cell-like cells resist chemotherapy but remain dependent on the EWSR1-WT1 oncoprotein
Source: Front Cell Dev Biol. 2022 Nov 25;10:1048709. doi: 10.3389/fcell.2022.1048709 (PMC9732033; doi:10.3389/fcell.2022.1048709)
Supplement: Supplementary file 1 [file DataSheet1.docx]

Supplementary Material

**Supplementary Table 1:** DSRCT Patient Tumor Samples

| **Patient** | **Primary Tumor Location** | **Age at Primary Tumor Harvest** | **Extraperitoneal Metastasis Location** | **Age Metastasis Harvest** | **Chemo-therapy?** |
| --- | --- | --- | --- | --- | --- |
| 1 | Peritoneum | 30 | Cervical LN | 32 | Yes |
| 2 | Peritoneum | 10 | Pleural implant | 11 | Yes |
| 3 | Peritoneum | Unknown | Mammary LN | Unknown | Yes |

**Supplementary Table 2:** RT-qPCR Primers

| **Gene** | **Fwd Primer (5’ -> 3’)** | **Rev Primer (5’ -> 3’)** |
| --- | --- | --- |
| ACTB | GCAAAGACCTGTACGCCAAC | AGTACTTGCGCTCAGGAGGA |
| NANOG | AGTCCCAAAGGCAAACAACCCACTTC | TGCTGGAGGCTGAGGTATTTCTGTCTC |
| POU5F1/OCT4 | GACAGGGGGAGGGGAGGAGCTAGG | CTTCCCTCCAACCAGTTGCCCCAAAC |
| SOX2 | GGGAAATGGGAGGGGTGCAAAAGAGG | TTGCGTGAGTGTGGATGGGATTGGTG |
| KLF4 | ACGATCGTGGCCCCGGAAAAGGACC | CAACAACCGAAAATGCACCAGCCCCAG |
| MYC | GCGTCCTGGGAAGGGAGATCCGGAGC | TTGAGGGGCATCGTCGCGGGAGGCTG |
| WT1 (c-term)/ EWSR1-WT1 | CCATACCAGTGTGACTTCAAGG | TGTGGGTCTTCAGGTGGTC |

**Supplementary Table 3:** Antibodies for Western Blot

| **Antibody** | **Company** | **Catalog #** | **WB Dilution** |
| --- | --- | --- | --- |
| β-Actin (8H10D10) | Cell Signaling | 3700 | 1:1000 |
| SOX2 (D6D9) | Cell Signaling | 3579 | 1:1000 |
| NANOG (D73G4) | Cell Signaling | 4903 | 1:500 |
| EWSR1 (N-term) | Lab Created | N/A | 1:1000 |
| Top IIa | Bethyl | A300-054A | 1:1000 |
| Top IIb | Bethyl | A300-949A | 1:1000 |


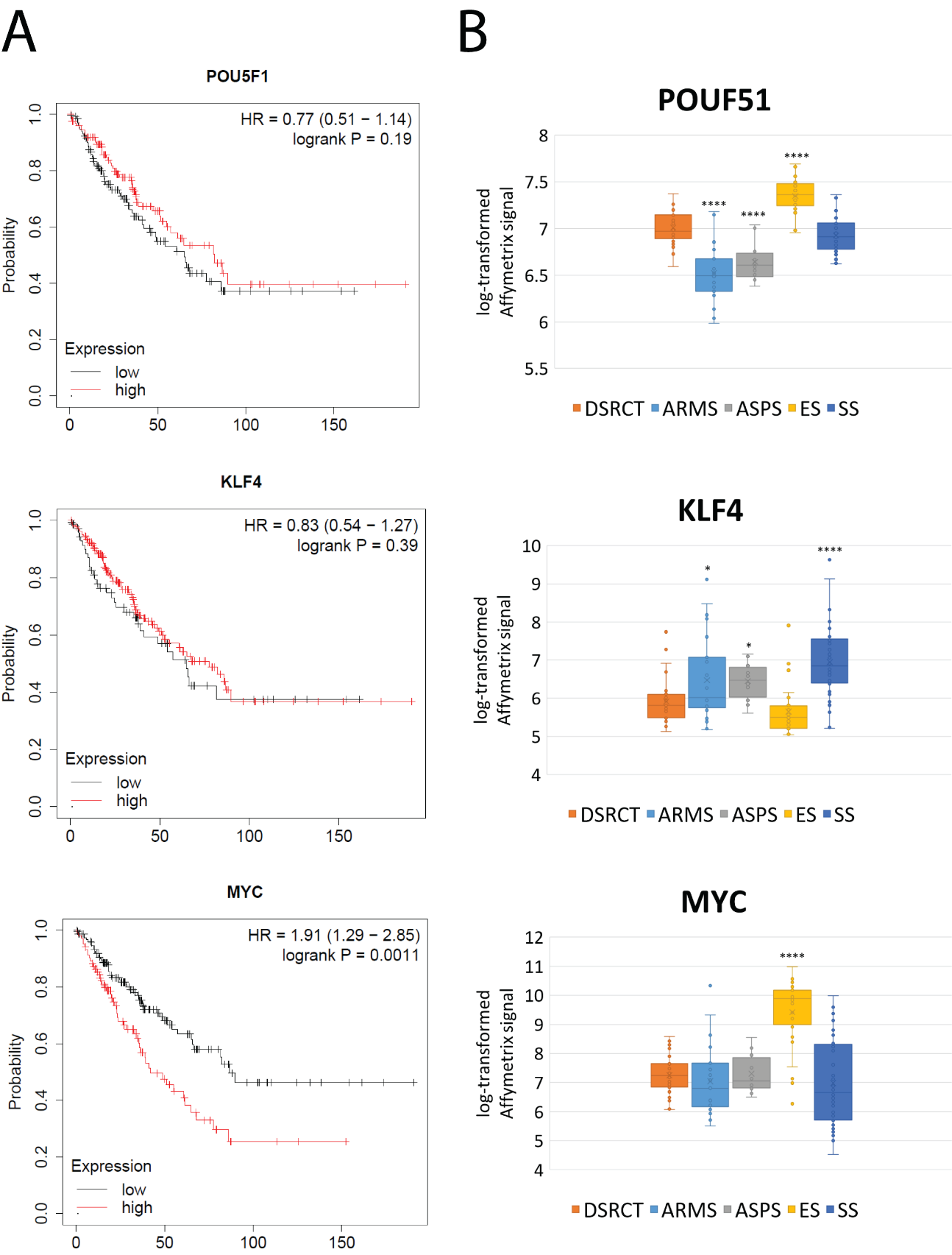


**Supplementary Fig 1. OCT4, KLF4, and MYC gene expression in DSRCT.** **(A)** Kaplan-Meier curve of sarcoma patient survival based on *OCT4 (POU5F1)* (High n=127, Low n=132)*, KLF4* (High n=186, Low n=73)*,* and *MYC* (High n=152, Low n=107) gene expression from KMplotter (n=259). **(B)** Relative transcript levels of *OCT4 (POU5F1), KLF4,* and *MYC* in DSRCT (n=28) ARMS (n=23), ASPS (n=12), ES (n=28), and SS (n=46) primary tumors based on Affymetrix U133A expression array data.


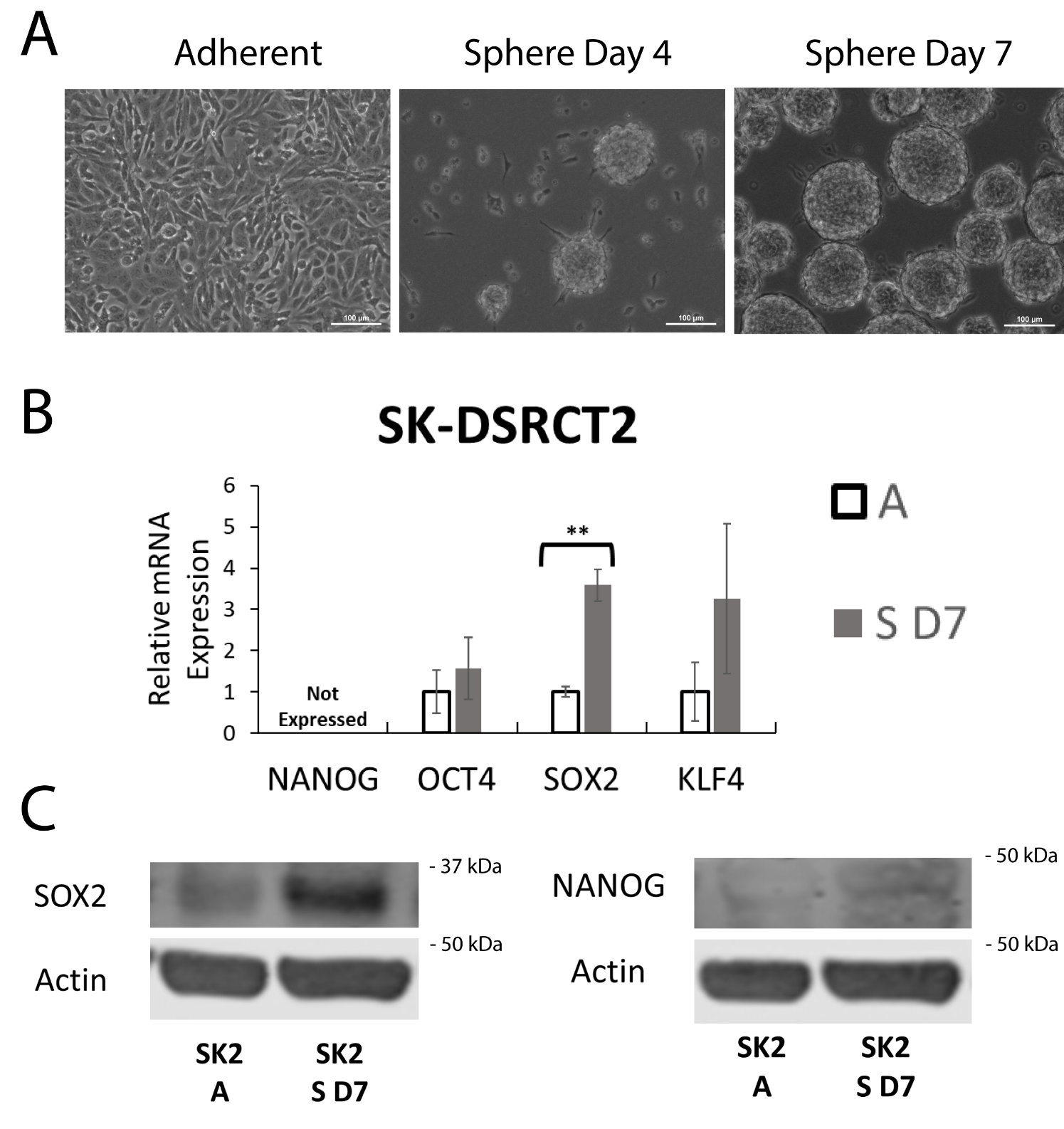


**Supplementary Fig 2. SK-DSRCT2 tumorsphere formation and stemness expression.** **(A)** Light microscopy of SK-DSRCT2 cells grown in adherent culture or sphere culture for 4 and 7 days. **(B)** Relative mRNA expression of NANOG, POU5F1, SOX2, KLF4, and MYC in adherent culture “A” or 7 days of sphere culture “S D7” assessed via RT-qPCR (n=3, * p<0.05, ** p<0.01, *** p<0.001, student t-test). **(C)** Western blot of *SOX2* and *NANOG* protein expression in adherent culture or 7 days of sphere culture.


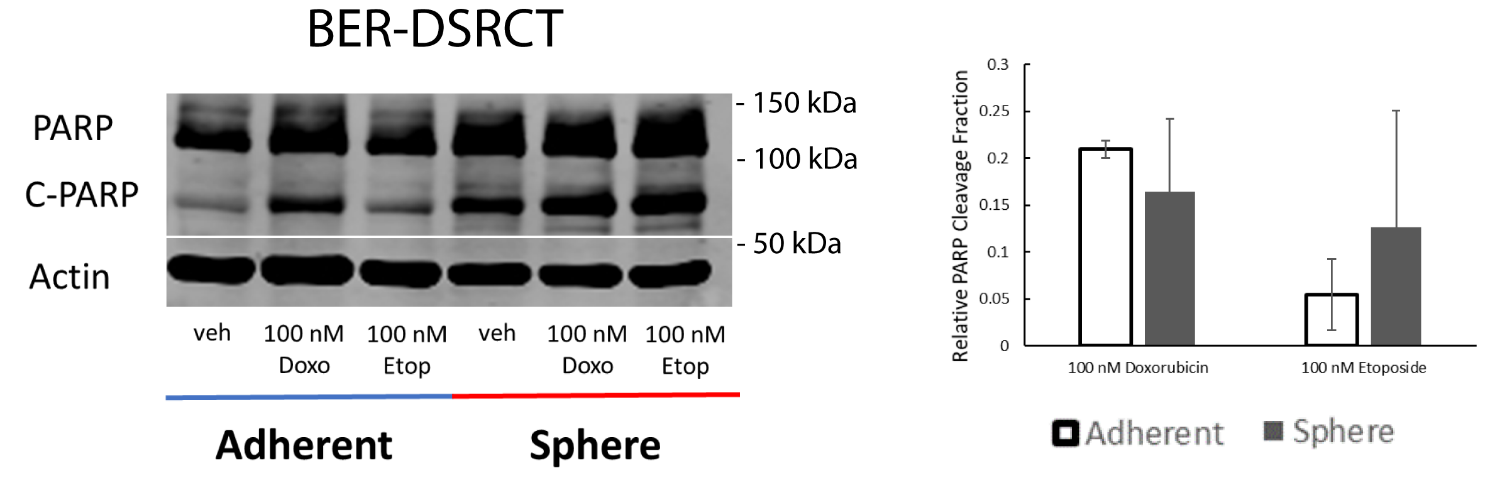


**Supplementary Fig 3. BER-DSRCT Chemoresistance.** Western blot of PARP and cleaved PARP after 24 hr treatment with vehicle, 100 nM doxorubicin, or 100 nM etoposide in adherent or sphere culture conditions in BER-DSRCT. Relative protein expression from three independent biological replicates quantified using ImageJ is shown in left panel (n=3 Error bars = STD).


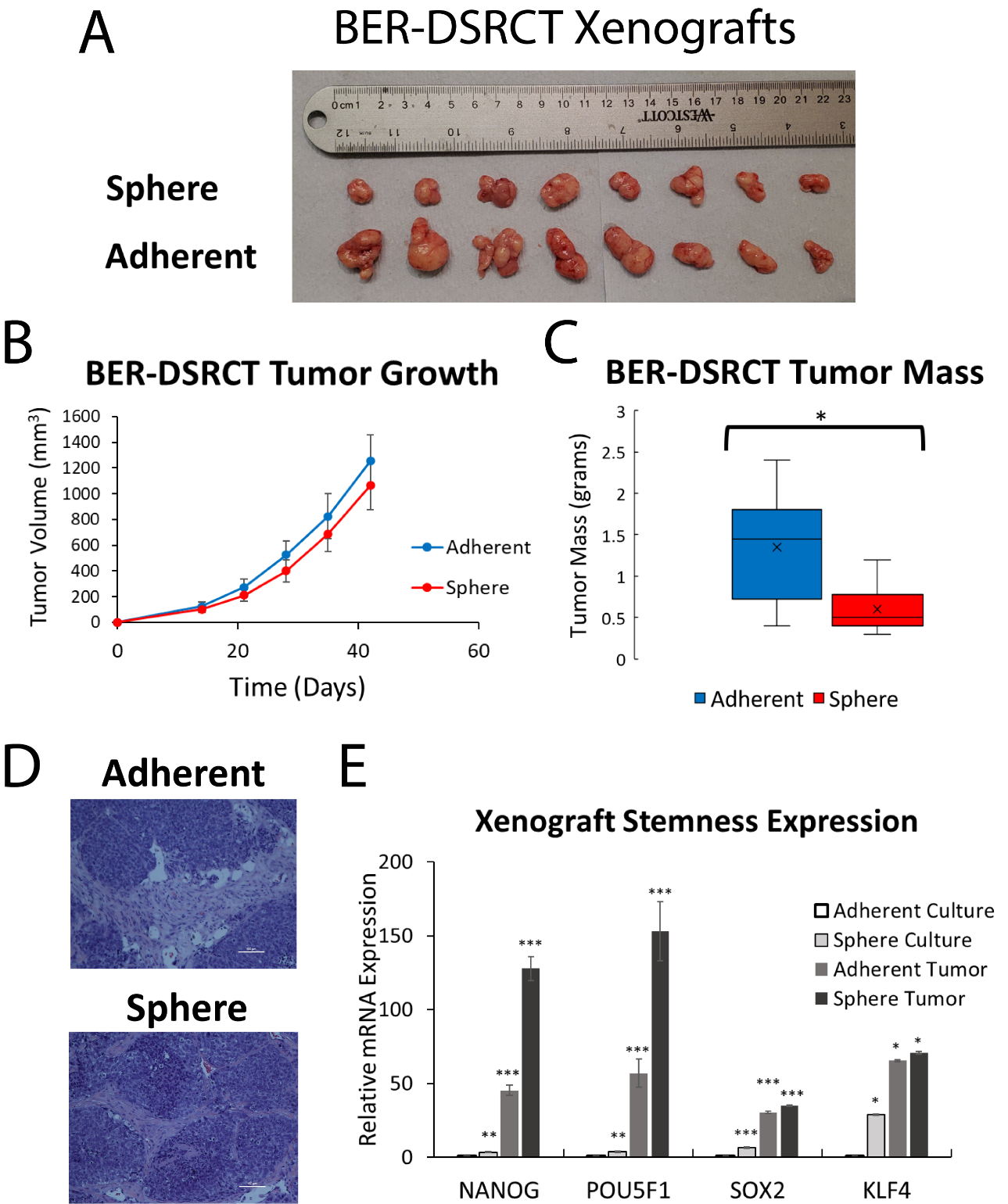


**Supplementary Fig 4. BER-DSRCT CSC-like cells form tumors in vivo.** **(A)** BER-DSRCT sphere and adherent culture cells form tumors in 8/8 xenograft injections. **(B)** Tumor volume of BER-DSRCT xenografts seeded from adherent or sphere culture cells. **(C)** Final mass of BER-DSRCT xenografts seeded from adherent or sphere culture cells (* p<0.05, student t-test). **(D)** Representative of H&E staining of BER-DSRCT xenografts seeded from adherent or sphere culture cells. **(E)** Relative mRNA expression of *NANOG, POU5F1, SOX2, and KLF4* in BER-DSRCT cells in adherent culture, sphere culture, xenograft tumors derived from adherent culture, or xenograft tumors derived from sphere culture assessed via RT-qPCR (n=3, * p<0.05, ** p<0.01, *** p<0.001, student t-test).


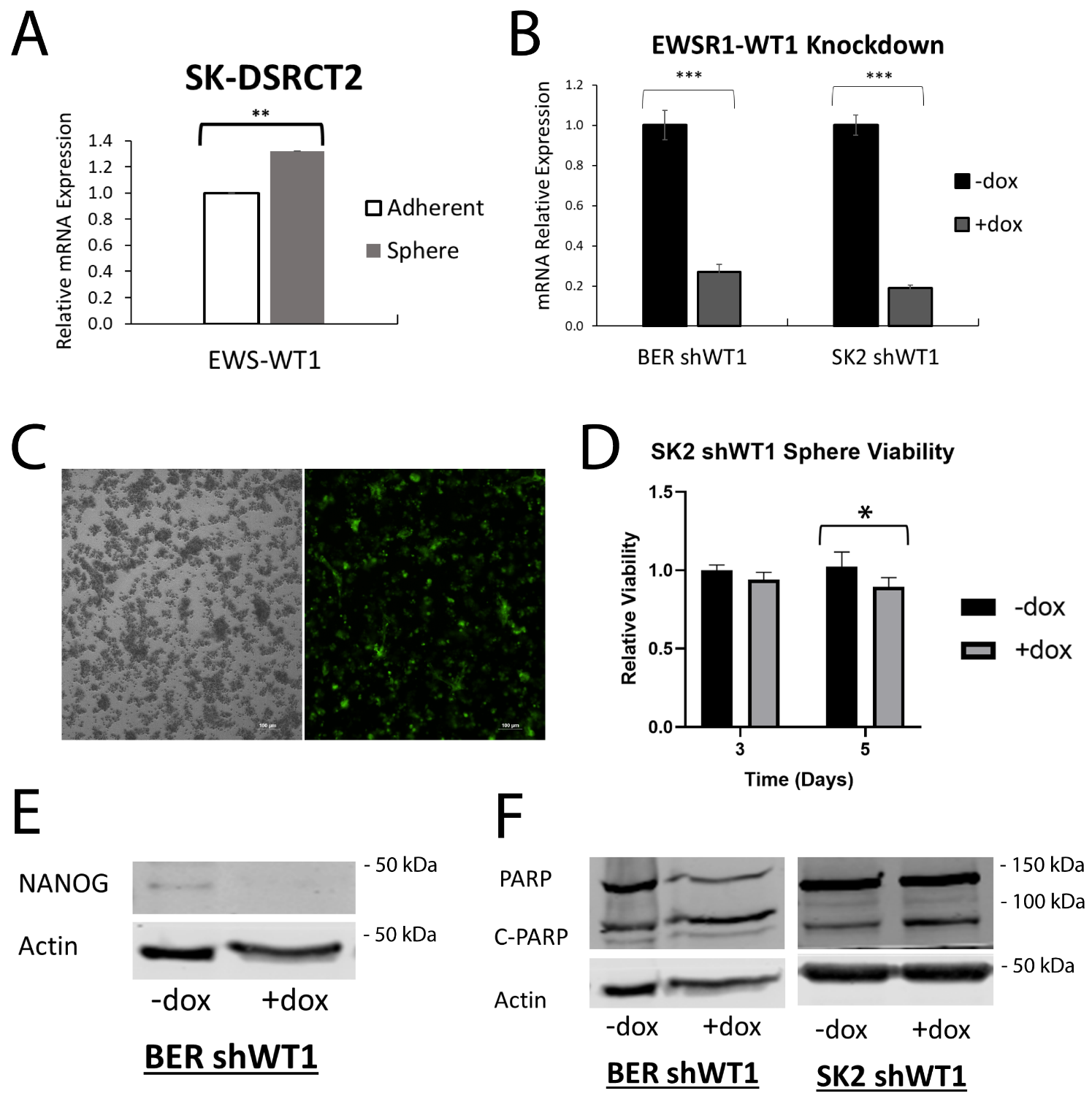


**Supplementary Fig 5. EWSR1-WT1 Knockdown in DSRCT CSCs.** **(A**) Relative mRNA expression of *EWSR1-WT1* in SK-DSRCT2 cells in adherent culture or 7 days of sphere culture assessed via RT-qPCR (n=3, ** p<0.01, student t-test). **(B**) Relative mRNA expression of *EWSR1-WT1* in BER-DSRCT shWT1 and SK-DSRCT2 shWT1 cells with or without dox assessed via RT-qPCR (n=3, *** p<0.001, student t-test). **(C)** Light and fluorescent microscopy of BER shWT1 cells that were induced to knockdown *EWSR1-WT1* after 4 days of sphere culture. Pictures were taken 5 days after dox induction. **(D)** Relative viability of SK-DSRCT2 shWT1 in sphere culture for 3 and 5 days with or without dox addition (n=4, * p<0.05, student t-test). **(E)** Western blot of NANOG protein expression in BER-DSRCT shWT1 cells in sphere culture 7 days with or without dox addition. **(F)** Western blot of PARP and cleaved PARP in BER-DSRCT shWT1 and SK-DSRCT2 shWT1 cells in sphere culture 7 days with or without dox addition.
